# William H. Prusoff (1920–2011): Father of Antiviral Chemotherapy

**DOI:** 10.1371/journal.pbio.1001190

**Published:** 2011-11-08

**Authors:** Yung-Chi Cheng

**Affiliations:** Yale School of Medicine, New Haven, Connecticut, United States of America

**Figure:**
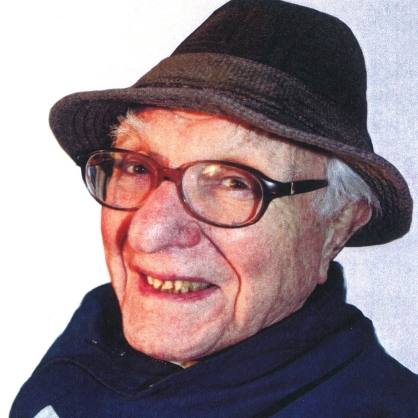
William H. Prusoff. Image credit: Laura Prusoff.

Regarded as the father of antiviral chemotherapy, William H. Prusoff (Bill) passed away on April 3, 2011, in New Haven, Connecticut. Dr. Prusoff spent most of his career studying analogs of thymidine, a nucleoside building block of DNA, with an eye toward developing therapeutic agents. By exploring analogs to thymidine for use as antiviral drugs, his research created a new scientific paradigm for antiviral drug development.

In the late 1950s, Bill synthesized one of the first thymadine analogs, 5-iododeoxyuridine. At the time, it was thought to be difficult to find antiviral drugs with a high therapeutic index, but Professor Herbert E. Kaufman found that the compound could be used as an effective topical treatment for herpes virus keratitis by disrupting the virus's ability to reproduce. More significantly, though, this discovery was a scientific game changer—it was the first time that a clinical antiviral drug had been shown to have selective antiviral activity if used properly.

My association with Bill started in the early 1970s during my postdoctoral work at Yale. Together with Professor David Ward, we found that 5′amino-5-iododeoxyuridine had a high degree of selectivity against the herpes simplex virus in culture. Around the same time, Dr. James Black and Dr. Gertrude Elion at Burroughs Wellcome found that acyclovir, another nucleoside analog, also exhibited anti-herpes virus behavior in culture. Scientifically, these were major breakthroughs because these two discoveries were the first demonstrations of highly selective antiviral drug behavior due to the unique properties of the herpes simplex virus. Subsequently, it was found that the selectivity was due to the preferential activation of compounds by viral specified thymidine kinase. Due to an unexpected toxicity found in young mice, 5′amino-5-iododeoxyuridine was never further developed, but acyclovirin went on to become the first orally active anti-herpes virus drug.

In the 1980s, while the AIDS epidemic was spreading and found to be caused by HIV, Bill and the late Dr. Tai-Shun Lin discovered that a compound synthesized by Dr. Jerome Horwitz had potent anti-HIV properties. The compound was originally named D4T, and Bristol-Myers Squibb developed and marketed this drug under its more common name, Zerit. It became a key drug as part of the first combination therapy for treating AIDS. Realizing that this treatment could provide great benefits to those struggling with the growing HIV crisis in impoverished Africa, Doctors Without Borders and Yale students later lobbied Yale University and Bristol-Myers Squibb to make Zerit available at a low cost for the African market. Bill quickly joined the effort, even though it meant a loss of personal income. “We are not doing this to make money, we are interested in developing a compound that would be a benefit to society,” he explained. The effort to make Zerit more affordable was a success: millions of people around the world benefited from Bill's research and humanitarian efforts.

Bill was born on June 25, 1920, in New York City and attended the University of Miami. After receiving his undergraduate degree in chemistry, he obtained his PhD from Columbia University and later completed his postdoctoral training in the laboratory of Professor Arnold Welch at Case Western Reserve University. After Dr. Welch was recruited by Yale to head the Medical School's pharmacology department, Bill was invited to join the same department as an assistant professor and was subsequently promoted to the rank of professor. This relationship at Yale would span over the next 58 years, with Bill becoming one of Yale's most well respected scientists and teachers.

Though Bill officially retired at age 70, he never stopped working and continued to be industrious. Until his death, his work as professor emeritus concentrated on the potential for using boronated-thymidine analogs as sensitizing cancer agents for neutron therapy.

Bill's remarkable contributions did not go unnoticed in his lifetime. Among his many accolades, he received the ASPET Award from the American Society of Pharmacology and Experimental Therapeutics and the Peter Parker Medal, Yale School of Medicine's highest award. He also received the Inaugural Lifetime Achievement Award from the Yale Comprehensive Cancer Center. His legacy was further solidified when the School of Medicine established an endowed chair in his name and the Department of Pharmacology named one of its conference rooms after him. In addition, the International Society for Virus Research established the William Prusoff Young Investigator Lecture Award.

Despite his numerous awards and prestigious accomplishments, Bill was an extremely humble and down-to-earth man who was quick to understate his achievements and even quicker to share the credit with others. He lived modestly, unselfishly, and with the highest integrity. These traits made him an extremely popular and highly respected individual everywhere. I do not remember a time that anyone would say anything bad about him. He was always eager to assist others. As a fellow at Pierson College here at Yale, he would provide students with help but never boasted or took credit. Bill also generously contributed to charities, endowing several lectureships in pharmacology, virology, and public health at Yale, supporting research in several laboratories, and establishing the William H. Prusoff Foundation to support various programs, including the United Way and the Yale Initiative for the Interdisciplinary Study of Anti-Semitism. He dedicated his life to the service of others because he saw generosity and helping as a natural extension of the human condition. He was a role model for scholars to follow.

Bill was always upbeat, smiling and ready with a joke. His lifelong optimism in the worst of times was always inspiring—he often said “the best is yet to come.” Even in the hospital under intense discomfort, he kept a lighthearted and positive disposition, making light of his situation when we knew how serious it was. He even kept on sending us jokes through email when he couldn't tell them in person.

For those of us fortunate enough to have personally known him, we were gifted with his peculiar sense of humor, warm welcoming company, and deep wisdom. Bill's influence was much broader, though: training a new generation of scientists who have gone on to become successful in their own right, rippling with their own contributions that have affected others. For the millions of lives his work has touched and saved, we have lost a giant scientist in this age.

